# Orthogonal P-wave morphology is affected by intra-atrial pressures

**DOI:** 10.1186/s12872-017-0724-x

**Published:** 2017-12-06

**Authors:** Richard Petersson, J. Gustav Smith, David A. Larsson, Öyvind Reitan, Jonas Carlson, Pyotr Platonov, Fredrik Holmqvist

**Affiliations:** 1 Department of Cardiology, Clinical Sciences, Lund University, Lund, Sweden; 2 Center for Integrative Electrocardiology, Lund University, Lund, Sweden; 3 Heart Failure and Valvular Disease Clinic, Skåne University Hospital, Lund, Sweden; 4 Department of Internal and Emergency Medicine, Skåne University Hospital, Lund, Sweden; 5 Arrhythmia Clinic, Skåne University Hospital, Lund, Sweden

**Keywords:** P-wave morphology, Atrial electrophysiology, Electrocardiography, Pressure, Right heart catheterization, Echocardiography

## Abstract

**Background:**

It has previously been shown that the morphology of the P-wave neither depends on atrial size in healthy subjects with physiologically enlarged atria nor on the physiological anatomical variation in transverse orientation of the left atrium. The present study aimed to investigate if different pressures in the left and right atrium are associated with different P-wave morphologies.

**Methods:**

38 patients with isolated, increased left atrial pressure, 51 patients with isolated, increased right atrial pressure and 76 patients with biatrially increased pressure were studied. All had undergone right heart catheterization and had 12-lead electrocardiographic recordings, which were transformed into vectorcardiograms for detailed P-wave morphology analysis.

**Results:**

Normal P-wave morphology (type 1) was more common in patients with isolated increased pressure in the right atrium while abnormal P-wave morphology (type 2) was more common in the groups with increased left atrial pressure (P = 0.032). Moreover, patients with increased left atrial pressure, either isolated or in conjunction with increased right atrial pressure, had significantly more often a P-wave morphology with a positive deflection in the sagittal plane (P = 0.004).

**Conclusion:**

Isolated elevated right atrial pressure was associated with normal P-wave morphology while left-sided atrial pressure elevation, either isolated or in combination with right atrial pressure elevation, was associated with abnormal P-wave morphology.

## Background

By analysing P-wave morphology, important information about the three-dimensional atrial activation may be acquired [1, 2]. For instance, abnormal P-wave morphology has been shown to be associated with the development of atrial fibrillation in patients with reduced left ventricular function [3].

Different orthogonal P-wave morphology types have previously been identified, likely corresponding to different atrial depolarization routes [1]. The difference between the types is mainly in the sagittal plane, in which P-waves may present as predominantly positive, biphasic or negative. The magnitude of the negative P-wave component in right precordial leads, and lead V_1_ in particular, has been named P-wave terminal force (PTF) and is included in the concept of the electrocardiographical left atrial (LA) abnormality [4].

Although one may speculate that factors other than electrical activation routes affect P-wave morphology, our group has previously shown that P-wave morphology does not depend on atrial size in healthy subjects with physiologically enlarged atria [5] and that the physiological anatomical variation in transverse orientation of the LA does not influence P-wave morphology [6]. The present study was primarily designed to investigate if different pressures in the LA and right atrium (RA) are associated with different orthogonal P-wave morphologies. Our hypothesis was that orthogonal P-wave morphology differs between patients with left- and right-sided atrial pressure elevations. To address this question, we chose to retrospectively study patients who had undergone right heart catheterization (RHC).

## Methods

### Right heart catheterization

Adult patients who underwent clinically motivated RHC at Skåne University Hospital in Lund between September 2000 and May 2013 were retrospectively screened for inclusion in the current study. Patients included in the study had undergone RHC because of pulmonary hypertension, heart failure where heart transplantation was considered, or valvular heart disease. Patients with Grown-Up Congenital Heart Disease (GUCH) and patients after heart transplantation were not included. All RHCs were performed as standard procedures at rest in the recumbent position, using Swan-Ganz catheters inserted mainly via the right internal jugular vein by the Seldinger technique [7]. Catheter position was confirmed by fluoroscopy. Continuous measurements were obtained for right atrial pressure and pulmonary arterial wedge pressure. Only first-time RHC procedures were considered.

The patients were classified as having a markedly elevated pressure in the LA, based on pulmonary arterial wedge pressure (PAWP), the RA, based on right atrial pressure (RAP), or both atria. The following definitions were used:

- Isolated, markedly elevated LA pressure: PAWP ≥15 mmHg and RAP ≤5 mmHg
- Isolated, markedly elevated RA pressure: PAWP ≤12 mmHg and RAP ≥8 mmHg
- Bi-atrial, markedly elevated pressures: PAWP ≥15 mmHg and RAP ≥8 mmHg

The upper limits for normal intra-atrial pressures used were 12 and 5 for PAWP and RAP, respectively [8]. The threshold for markedly elevated pressure was set higher than the upper limit of the normal range to ensure only patients with pathologically increased pressures were included. Patients not meeting these criteria and patients lacking data on either PAWP or RAP were excluded.

### Echocardiography

We retrospectively collected the echocardiography images closest to the date of the RHC. For quantification of RA size, we used end-systolic area from the apical four-chamber view [9]. LA end-systolic volume was calculated using the area-length technique in the apical two- and four-chamber views. LA end-systolic area and single plane volume estimations are also presented. Chamber quantifications were indexed to body surface area (BSA), according to recommendations [10]. The normal upper values for RA and LA area used were 11.0 cm^2^/m^2^ and 11.8 cm^2^/m^2^, respectively [9]. All measurements were made in Philips Xcelera R4.1 L1 4.1.1.1133–2013 running on Microsoft Windows 7. All echocardiograms were clinically motivated examinations.

### ECG acquisition and analysis

**Fig. 1:**
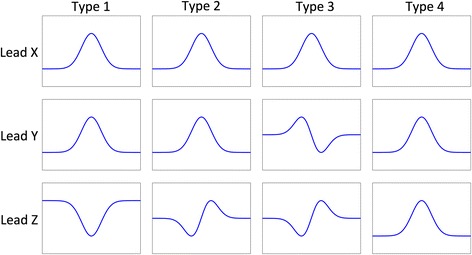
Type 1: predominantly positive leads X and Y and predominantly negative lead Z, type 2: predominantly positive leads X and Y and biphasic lead Z (negative, positive), type 3: predominantly positive lead X and biphasic leads Y (positive, negative) and Z (negative, positive) and type 4 (positive in all three leads)

From the two major ECG databases in use in Skåne Region (Infinity MegaCare and GE Healthcare MUSE), we digitally retrieved the 12-lead ECG closest to a patient’s RHC. The ECG was considered obsolete if it had not been recorded within two months of the catheterization. All ECGs were analysed using custom-built software running on MATLAB R2013B for Mac OS X (The MathWorks, Inc., Natick, MA, USA). All ECGs were transformed into derived vectorcardiograms using the inverse Dower transform [11]. A 0.5-Hz high-pass filter and a 50-Hz band-stop filter were then applied. QRS complexes were automatically identified and grouped together using a cross-correlation coefficient of *ρ* > 0.9. A time-window of 250 ms preceding the QRS complex was used to extract the P-wave. In cases with a long PQ time, this time-window was manually adjusted. The P-waves were time-shifted to achieve maximum correlation and were clustered together based on a cross-correlation coefficient of *ρ* > 0.9. P-waves belonging to the same cluster were then signal-averaged. The beginning and end of the P-wave were manually defined. The method has been described in more detail elsewhere [12]. The signal-averaged P-waves were classified as one of four types or “atypical” (Fig. 1) [2, 12].

All ECGs were also automatically analysed regarding PTF, which was defined as the product of the duration and the absolute value of the amplitude of the terminal, negative part of the P-wave in lead V_1_ (mm × s) in cases with a biphasic, positive/negative P-wave [13]. A PTF of more than 0.04 mm × s is considered a sign of LA abnormality, including but not limited to elevated LA pressure [4].

### Statistics

Normally distributed data are presented as mean ± standard deviation (SD), otherwise as median and quartiles. Student’s t-test was used for comparing two groups of normally distributed data. When comparing more than two groups, One-Way ANOVA and Kruskal-Wallis were used for parametric and nonparametric data, respectively. Pearson’s chi-squared test was used for comparing groups of nominal data. For correlation analysis, Pearson’s correlation coefficient, *r*, and the coefficient of determination, *R* ^2^, were computed. ROC (receiver operating characteristic) curve and area under the curve were used to evaluate sensitivity and specificity. Differences were considered significant when *P* < 0.05. All statistical analyses were done using IBM SPSS Statistics version 23 running on Mac OS X.

## Results

**Fig. 2:**
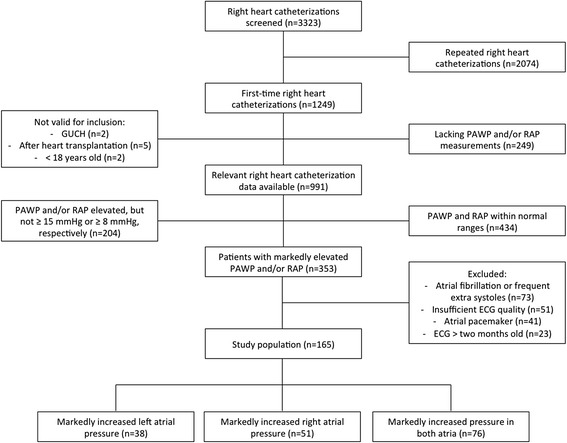
Flow chart for inclusion and exclusion of study participants

Between September 2000 and May 2013, 3323 RHCs had been performed, of which 1249 were first-time procedures. Figure 2 presents an overview of patient inclusion and exclusion. After exclusion, 165 patients were eligible and distributed as follows: 38 patients with isolated, increased LA pressure, 51 patients with isolated, increased RA pressure and 76 patients with biatrially increased pressure.

There were 25 patients with P-wave morphology type 1, 99 with type 2, six with type 3 and 34 with type 4. One patient did not fall into any of the P-wave morphology classes and was classified as “atypical”.

The distribution of P-wave morphology was significantly different depending on intra-atrial pressures. P-wave morphology type 1 was more common in patients with isolated increased pressure in the RA than in the other groups (*P* = 0.032). P-wave morphology type 2 was more common in the groups with increased LA pressure, as presented in Table 1. Patients with increased LA pressure, either isolated or in conjunction with increased RA pressure, had significantly more often a P-wave morphology with a positive deflection in lead Z – either type 2 (biphasic negative/positive) or type 4 (positive) (Table 2).

**Table 1:** P-wave morphology type and intra-atrial pressures

		Markedly elevated intra-atrial pressure		
	All (*n* = 165)	Left (*n* = 38)	Right (*n* = 51)	Both (*n* = 76)
Type 1	25 (15%)	4 (11%)	14 (28%)	7 (9%)
Type 2	99 (60%)	23 (61%)	28 (55%)	48 (63%)
Type 3	6 (4%)	1 (3%)	0	5 (7%)
Type 4	34 (21%)	10 (26%)	8 (16%)	16 (21%)
Atypical	1 (1%)	0	1 (2%)	0

*P* = 0.06 when comparing all morphology types. P = 0.032 when comparing only types 1 and 2. Pearson’s chi-squared test

**Table 2:** Markedly increased pressure in the LA (isolated or biatrial) vs. normal LA pressure in regard to the deflection of the P-wave in lead Z – negative (type 1) or biphasic/positive (type 2 or type 4)

	Normal LA pressure	Markedly elevated LA pressure	Total
Type 1 (negative Z)	14 (28%)	11 (10%)	25
Type 2 and 4 (biphasic or positive Z)	36 (72%)	97 (90%)	133
Total	50	108	158

P = 0.004 (Pearson’s chi-squared test). *LA* Left atrial

The median number of days between the RHC and the echocardiographic examination was 3 days with a lower and upper quartile of 1 and 12, respectively.

LA dimensions were significantly greater in patients with increased LA pressure than in patients with isolated elevated RA pressure (e.g., LA volume/BSA 52.4 ± 13.5 ml/m^2^ vs. 22.4 ± 11.7 ml/m^2^, *P* < 0.001). Conversely, in patients with isolated increased RA pressure, the RA area/BSA was increased (15.1 ± 5.0 cm^2^/m^2^ vs. 10.6 ± 4.4 cm^2^/m^2^, *P* = 0.001), as shown in Table 3.

**Table 3:** Patient characteristics

		Markedly elevated intra-atrial pressure			
	All (*n* = 165)	Left (*n* = 38)	Right (*n* = 51)	Both (*n* = 76)	*P*
Age (*n* = 165)	54 ± 16	51 ± 14	59 ± 17	52 ± 15	*0.02*
Male (*n* = 165)	90 (55%)	26 (68%)	21 (41%)	43 (57%)	*0.03*
Diastolic blood pressure (mmHg) (*n* = 152)	75 ± 13	73 ± 11	79 ± 13	73 ± 13	*0.01*
Mean blood pressure (mmHg) (*n* = 152)	90 ± 15	86 ± 13	95 ± 15	86 ± 14	*< 0.01*
Systolic blood pressure (mmHg) (*n* = 152)	117 ± 24	113 ± 21	127 ± 24	113 ± 23	*< 0.01*
PAWP (mmHg) (*n* = 165)	*17 ± 7*	*18 (16, 21)*	*8 (6, 10)*	*22 (17, 25)*	
RAP (mmHg) (*n* = 165)	*10 ± 6*	*3 (2, 5)*	*11 (9, 14)*	*12 (9, 16)*	
Height (m) (*n* = 165)	1.72 ± 0.11	1.75 ± 0.12	1.69 ± 0.11	1.72 ± 0.11	*< 0.05*
Weight (kg) (*n* = 165)	79 ± 19	83 ± 23	77 ± 17	80 ± 17	0.27
BMI (kg/m^2^) (*n* = 165)	26.8 ± 5.2	26.8 ± 5.3	26.8 ± 5.4	26.9 ± 5.1	0.99
P-wave duration (ms) (*n* = 165)	136 ± 18	144 ± 19	132 ± 14	136 ± 18	*0.01*
PQ time (ms) (*n* = 165)	172 ± 28	177 ± 29	170 ± 25	171 ± 30	0.42
Diagnoses					
- PH	49 (30%)	1 (3%)	40 (78%)	8 (11%)	*< 0.001*
- HF	109 (66%)	33 (87%)	12 (24%)	64 (84%)	*< 0.001*
- Valvular	8 (5%)	4 (11%)	0 (0%)	4 (5%)	0.07
Echo					
- LA volume/BSA (ml/m^2^) – A4C and A2C area length (*n* = 57)	45.6 ± 18.6	52.4 ± 13.5	22.4 ± 11.7	49.5 ± 17.0	*< 0.001*
- LA area/BSA (cm^2^/m^2^) A4C (*n* = 123)	11.9 ± 4.3	13.4 ± 4.0	8.2 ± 3.0	13.4 ± 3.9	*< 0.001*
- LA volume/BSA (ml/m^2^) – A4C single plane (*n* = 123)	38.3 ± 21.5	47.8 ± 21.4	19.8 ± 12.8	45.0 ± 18.9	*< 0.001*
- RA area/BSA (cm^2^/m^2^) – A4C (*n* = 112)	12.5 ± 4.5	10.6 ± 4.4	15.1 ± 5.0	11.7 ± 3.3	*< 0.001*

Mean ± standard deviation and median (lower and upper quartiles). Analysed with One-Way ANOVA, Kruskal-Wallis and Pearson’s chi-squared test. *P*-values in italic are significant (*P* < 0.05). *PH* Pulmonary hypertension (classified in medical records as primary or secondary pulmonary arterial hypertension and chronic thromboembolic pulmonary hypertension), *HF* Heart failure (classified as cardiomyopathy [dilated, hypertrophic, ischemic and unspecified], constrictive pericarditis and unspecified heart failure). One patient may have several diagnoses. *LA* Left atrial, *RA* Right atrial, *BSA* Body surface area, *A4C* Apical 4-chamber view, *A2C* Apical 2-chamber view

Patients with isolated increased RA pressure were significantly older, more often female and had higher blood pressure compared to patients with elevated LA pressure. Patients with isolated increased LA pressure had significantly longer P-wave duration compared to the other groups. The mean height was found to be borderline significantly greater in patients with isolated increased LA pressure than in the other groups. The predominant diagnosis in patients with elevated RA pressure was pulmonary hypertension and the major diagnosis of patients with elevated pressure on the left side was heart failure, as shown in Table 3.

When looking only at patients with a LA area within the normal range, P-wave morphology type 2 was overrepresented in patients with isolated increased LA pressure (Table 4). In patients with normal RA area there was no significant difference in the distribution of P-wave morphologies.

**Table 4:** Patients with normal left atrial size (LA area/BSA ≤ 11.8 cm^2^/m^2^). Only P-wave morphologies 1 and 2

		Markedly elevated intra-atrial pressure	
	All (*n* = 34)	Left (*n* = 7)	Right (*n* = 27)
Type 1	11 (32%)	0	11 (41%)
Type 2	23 (68%)	7 (100%)	16 (59%)

*P* = 0.04 (Pearson’s chi-squared test). *LA* Left atrial, *BSA* Body surface area. Isolated increased pressure in the left atrium was associated with P-wave morphology type 2 also when the left atrial size was normal

Of the 165 patients, 63 (38%) had a PTF greater than 0.04 mm × s, as shown in Table 5. In a correlation analysis between LA pressure and PTF, we found a positive correlation of *r* = 0.18 and an *R* ^2^ of 0.03 (*P* = 0.02). A sensitivity and specificity analysis of PTF for identifying markedly elevated LA pressure was conducted using a ROC curve. This yielded an area under the curve of 0.61.

**Table 5:** PTF vs. LA pressure

	PTF ≤ 0.04 mm × s	PTF > 0.04 mm × s	Total
Normal LA pressure	36 (71%)	15 (29%)	51
Markedly elevated LA pressure	66 (58%)	48 (42%)	114
Total	102	63	165

*P* = 0.121 (Pearson’s chi-squared test). *LA* left atrial, *PTF* P-wave terminal force in lead V_1_

## Discussion

We found that P-wave morphology was associated with intra-atrial pressure. Isolated increased pressure in the RA was associated with a higher prevalence of P-wave morphology type 1, while increased pressure in the LA or both atria was associated with a higher prevalence of type 2 P-wave morphology. Also, an increased LA pressure was strongly associated with a P-wave morphology with a positive deflection in lead Z (either type 2 or 4).

P-wave morphology type 1 is usually considered the “healthier” P-wave morphology and is commonly seen in young people [14]. The current finding that isolated elevated RA pressure was associated with P-wave morphology type 1 should be interpreted with caution since we do not have a group with healthy subjects to compare with. Hence, we do not know the distribution of P-wave morphologies in healthy subjects with normal intra-atrial pressures. All subjects in the current study had been referred to a RHC for clinical reasons. Therefore, the subjects with normal intra-atrial pressures could not be considered healthy and were hence not suitable for analysis. One may speculate that the elevated pressure in the RA potentiates the initial part of the P-wave, reflecting in a negative deflection in lead Z. It may also be that P-wave morphology type 1 is more common when the LA pressure is not elevated. This would explain why type 1 is more common in healthy subjects as well as in patients with isolated elevated RA pressure.

In the absence of LA dilatation, P-wave morphology type 2 was still overrepresented among patients with isolated, increased LA pressure. For this analysis, LA area and not volume was used because of sparse LA volume data availability. In a study by Morris et al., there was a significant relationship between PTF and LA pressure [13]. Likewise, Kasser et al. found that PTF was reliable at identifying increased LA pressure [15]. A biphasic P-wave in lead V_1_, associated with abnormal PTF, would roughly correspond to a biphasic Z lead (negative, positive) and be in keeping with our findings. One may speculate that an increased intra-atrial pressure and dilatation deteriorate atrial conduction, which may be reflected as abnormal atrial activation. Di Bianco et al. found that a normal PTF excluded a PAWP of more than 24 mmHg and an abnormal PTF excluded a PAWP of less than 10 mmHg in a population of male patients requiring hemodynamic monitoring at a medical intensive care unit [16]. We could not reproduce this finding. In our study, patients with normal PTF had a PAWP of 2–35 mmHg and patients with abnormal PTF had a PAWP of 5–31 mmHg. Our study participants were more numerous (*n* = 165 vs. *n* = 61), not male only and had a broader range of diagnoses; in Di Bianco’s study the vast majority had coronary artery disease. We did, however, find a positive, but weak, correlation between PTF and LA pressure. In the ROC curve analysis, PTF performed poorly at identifying markedly increased LA pressure. The lack of a strong association between abnormal PTF and elevated LA pressure implies that P-wave morphology does not simply reflect atrial size and hemodynamics but is also greatly influenced by atrial conduction properties, e.g., atrial fibrosis.

A caudocranial activation of the LA is seen in advanced interatrial block, which is defined as a wide (≥ 120 ms) and biphasic (positive/negative) P-wave in inferior leads [17]. In the current study, all patients with advanced interatrial block (*n* = 6) also had type 3 P-wave morphology.

Other investigators have looked at other aspects of the P-wave and pressures obtained at RHC. Wokhlu et al. have shown that the P-wave amplitude in lead II is positively correlated to mean pulmonary artery pressure. They proposed that the propagated elevated pressure in the RA triggers atrial enlargement and increased P-wave amplitude in inferior leads [18]. Analogue to this, Henkens et al. showed a linear correlation between P-wave amplitude in lead II and pulmonary vascular resistance [19]. In the present study, pulmonary artery pressure and pulmonary vascular resistance were not investigated.

The observed longer P-wave duration in patients with increased LA pressure is in keeping with the previously described positive correlation between pulmonary capillary wedge pressure and P-wave duration by Faggiano et al. [20]. Similar findings were reported by Kishima et al. [21]. Several explanations have been proposed. One is that increased pressure leads to increased dispersion of effective refractory periods, which slows conduction and hence prolongs P-wave duration [22].

As expected, there was a significant difference in atrial dimensions between the groups with different intra-atrial pressures. Patients with increased pressure in the LA or both atria had a larger LA while patients with isolated increased pressure in the RA had a larger RA. Geske et al. showed a positive correlation between LA volume on echocardiography indexed to BSA and LA pressure [23]. A similar association for the right side was described by Sato et al., who found a correlation between maximum RA volume on cardiac magnetic resonance imaging indexed to BSA and RAP [24].

Orthogonal P-wave morphology may prove to be a valuable, non-invasive marker of increased LA pressure. ECG is widely available and could potentially be used to non-invasively monitor patients with elevated atrial pressures. Further studies are needed to explore this possible application.

### Limitations

The current study was retrospective by design, which is why some data is missing in some cases. The study does not include a control group with healthy subjects. The distribution of P-wave morphologies in healthy subjects with normal intra-atrial pressures is therefore unknown. The echocardiographic examinations and RHCs were not carried out simultaneously and the hemodynamic conditions may therefore have changed. Comparisons should therefore be interpreted with caution.

## Conclusions

Isolated elevated RA pressure was associated with P-wave morphology type 1 while left-sided atrial pressure elevation, either isolated or in combination with RA pressure elevation, was associated with P-wave morphology type 2. The exact mechanism needs further investigation.

## Abbreviations

BSA: Body surface area
GUCH: Grown-up congenital heart disease
LA: Left atrium/atrial
PAWP: Pulmonary arterial wedge pressure
PTF: P-wave terminal force
RA: Right atrium/atrial
RAP: Right atrial pressure
RHC: Right heart catheterization
ROC: Receiver operating characteristic
SD: Standard deviation
